# The non-coding RNAs of the H19-IGF2 imprinted loci: A focus on biological roles and therapeutic potential in Lung Cancer

**DOI:** 10.1186/s12967-015-0467-3

**Published:** 2015-04-09

**Authors:** Imad J Matouk, David Halle, Michal Gilon, Abraham Hochberg

**Affiliations:** Department of Biological Chemistry, Institute of Life Sciences, The Hebrew University of Jerusalem, Jerusalem, 91904 Israel

**Keywords:** H19, miR-675, IGF2, Oncomirs, Lung cancer, Imprinted cluster

## Abstract

Since it was first described, the imprinted cluster 11p15.5 has been reported to be deregulated in a variety of pediatric and adult cancers including that of the lung. Both protein coding and non-coding genes functioning as oncogenes or as tumor suppressor genes reside within this cluster. Oncomirs that can function as oncogenes or as tumor suppressors have also been reported. While a complete account of the role played by the 11p15.5 imprinted cluster in lung cancer is beyond the scope of this review, we will focus on the role of the non-coding RNAs processed from the *H19-IGF2* loci. A special emphasis will be given to the *H19/miR-675* gene locus. Their potential diagnostic and therapeutic use in lung cancer will be described.

## Introduction

Lung cancer is the leading cause of cancer death among men and women. The prognosis of most lung cancer patients, afflicted with either non-small-cell lung cancer (NSCLC) or small cell lung cancer (SCLC), is dismal. Survival is low; the poor U.S. survival rate of 15% at 5 years is even lower in Europe and rest of the world. The unfavorable outcome is mainly due to our relatively limited understanding of the molecular pathogenesis of this cancer. A greater knowledge of lung cancer’s molecular processes may yield accurate new biomarkers for this disease and lead to its molecular diagnosis and risk stratification. For a long time, chemotherapy has been the main treatment. Classic chemotherapy was designed to block cellular replication, mainly by interrupting DNA synthesis and mitotic division. However, the clinical outcome of this approach has not provided reasons for optimism [1]. Recent advances in the understanding of the molecular aspects of the process of tumorigenesis have allowed identification of certain molecular targets for cancer treatment. Currently classic chemotherapy is being combined with tyrosine kinase inhibitors, such as erlotinib and gefitinib, or with monoclonal antibodies such as bevacizumab.

A meta-analysis of the impact of bevacizumab treatment found that an overall survival benefit in lung cancer was seen [2]; however the overall survival was still low. This frustrating state-of-the–art emphasizes the importance of the search for innovative treatments. Translational research was at first to a large extent based on oncogenes, tumor suppressor genes and their products. Later on, growth signal transduction, mediated by specific membrane receptors, moved to the forefront of such research. Only recently, long non-coding RNA (lncRNA) and microRNA (miRNA) genes have started to get a wider recognition as an important part of the molecular origin of cancer [3-5].

### The emerging roles of non-coding RNA (ncRNA) genes in lung cancer

While this section is not meant to be a comprehensive list of the roles of ncRNAs in lung cancer, we will use specific examples to illustrate the way that lncRNA’s generalized modes of action can occur via diverse mechanisms.

While less than 2% of our DNA is translated, the vast majority of the genome is actively transcribed producing various types of ncRNA, including the long RNAs (>200 nucleotides) and miRNA.

The function of lncRNAs is the least understood aspect of ncRNA research. Many thousands of lncRNAs are produced from our genome, yet relatively few have well documented roles in tumorigenesis and even fewer have been reported to have roles in lung cancer. Examples of lncRNA genes having roles in lung tumorigenesis are H19, metastasis associated lung adenocarcinoma transcript 1 (MALAT1), smoke and cancer-associated lncRNA-1 (SCAL1), lncRNA-LET, intronic ncRNA AK126698, BC-200 and hox transcript antisense intergenic RNA (HOTAIR) [6-11].

LncRNA genes have developmental and tissue specific expression patterns and aberrant regulation in a variety of diseases, including cancer. lncRNAs regulate the expression either of their neighbouring genes in *cis*, or of more distant genes in *trans*.

Some lncRNAs have been associated with a spectrum of biological processes including epigenetics and chromatin modifications. HOTAIR is perhaps a relatively well understood example. HOTAIR can bind to polycomb repressive complex 2 (PRC2) and thus regulate gene expression by directing the polycomb protein group to target DNA regions, inducing changes in histone marks and chromatin structure and ultimately suppressing transcriptional activity [12]. Forced expression of HOTAIR enhances lung cancer cell migration and anchorage-independent cell growth and is associated with short disease-free survival in NSCLC [13]. Furthermore, HOTAIR contributes to cisplatin resistance of human lung adenocarcinoma cells via down-regulation of p21(WAF1/CIP1) expression [14].

Other lncRNAs perform their function, at least in part, by being precursors for miRNA. Several miRNAs have been reported to be hosted in the exons or introns of some lncRNA [15,16], including also imprinted lncRNA [17]. In a study aimed to identify novel imprinted genes Hagan et al., identified at least ten genes define the imprinted DlK1-Dio3 cluster located on mouse chromosome 12qF1. Seven imprinted non-coding RNAs with maternal expression (Meg3/Gtl2, Anti-peg11, Meg8, Irm/“Rian”, AK050713, AK053394 and Meg9/Mirg) have being identified, the majority of them contain miRNA and/ or snoRNAs in their introns as do their human orthologs [17]. The H19 lncRNA can serve also as a precursor for a miRNA (miR-675), and will be discussed in later sections [18]. Furthermore, Xia *et al.* have demonstrated recently that some lncRNAs can regulate gene expression and thus contribute to oncogenesis by acting as competing endogenous RNA or molecular sponges for miRNA thus having the potential to scavenge miRNA targets. Eight lncRNAs including H19 were identified as using this mode of action [19]. In addition some lncRNAs can function as antisense transcripts. Several sense/antisense pairs have been identified in lung cancer and are significantly associated with multiple clinopathological factors. Two such examples are NKX2-1-AS1/TTF1, and DSCAM-AS1/DSCAM [20].

Other lncRNAs can regulate alternative splicing, cell cycle regulation and nuclear import [21], in addition to being associated with structural components as precursors to small RNAs and even as regulators of mRNA decay [22].

lncRNAs can act also in *cis*; for example allelic imprinting depends on lncRNAs that mediate the silencing of neighboring genes. H19 lncRNA also belongs to this category [23].

Furthermore lncRNA expression may provide clinical information regarding disease etiology and so could be used in diagnostic tests. In lung cancer, a recent study identified 72 lncRNAs that are associated with tumor subtypes [24]. The characterization of such RNA species, from function to clinical applicability, is of major biological and clinical importance.

Several recent studies have examined the association between lncRNA expression and cancer. A group of lncRNAs having differential expression in NSCLC tumors and in normal tissue has been identified. Upregulation of lncRNA was found to be strongly correlated with poor prognosis and high invasion and migration activity [25]. A group of 111 lncRNAs termed lung cancer-associated lncRNA (LCALs) have been identified that show differential expression in both adenocarcinoma and squamous cell carcinoma when compared with matched controls [26]. Interestingly, the expression levels of many LCALs have significant associations with the mutational status of key oncogenes in lung cancer. The function of the majority of these LCALs is yet to be elucidated.

MiRNAs are short (21–23 nucleotides) single-strand non-coding RNA molecules whose important role in neoplasia has unfolded over recent years. A miRNA molecule exerts negative control on mRNA by annealing to a complementary sequence, which results in protein translation inhibition or mRNA degradation [27]. A miRNA can assume the role either of a tumor suppressor gene (TSG) when it targets an oncogene, or that of an oncogene, if it’s critical target is a TSG.

Important gene products playing a pivotal role in lung cancer have been reported to be targets of miRNAs. Let-7, miR-34 families, and miR-17-92 among others have already been shown to play important roles in lung cancer [28].

One of the miRNAs with a role in the pathogenesis of lung cancer is let-7. The RAS oncogene is targeted by members of the let-7 miRNA family, which are often under expressed or deleted in lung cancer, thus facilitating RAS over expression. Let-7 family members have recently been shown to be oncofetal miRNAs, playing a role both during human lung development and in its neoplastic counterpart. Kumar and his group have shown that let-7 functionally inhibits non-small cell tumor development. In addition they have reported that overexpression of let-7 g caused significant growth reduction in human non-small cell lung tumors [29]. Another report has shown that let-7c inhibits invasion and migration of human NSCLC by targeting ITGB3 and MAP4K3. This study measured the expression of let-7 members in NSCLC tumors and a significant association was noticed between low levels of let-7c expression and patients with metastasis [30].

Another miRNA family of relevance to lung cancer is miR-29, which is also under expressed in this malignancy. When expression is induced under experimental conditions by blocking specific methyltransferases, normal patterns of DNA methylation are restored and previously silenced TSG such as *FHIT* are now expressed [31].

Low expression levels of miR-34a (a transcriptionally induced target for p53) has been reported in lung cancers as well as in many other types of cancers, and low expression level correlate with high probability of relapse in NSCLC patients. Therapeutic approaches to the treatment of NSCLC based on miRNA are in pre-clinical development. Systemic delivery of the synthetic tumor suppressor miR-34 in a lipid-based vehicle in an approach called miRNA replacement therapy blocks tumor growth in mouse models, was well tolerated and did not induce an immune response indicating the safety of this approach [32]. This approach has also proved successful for let-7 replacement in reducing tumor growth in mouse models of lung cancer [33].

### 11p.15.5 miRNAs and lung cancer

To the best of our knowledge, three miRNAs processed from the 11p15.5 cluster have been discovered to date, namely miR-675 and miR-483 belonging to the *H19-IGF2* loci, and miR-210. While each of these miRNAs has its own targets, it is noticeable that all of them are modulated by hypoxic stress, a common feature of the tumor microenvironment, and contribute to the repression of biologically important genes. It is possible that 11p15.5 miRNAs contribute to tumor progression by accommodating cancer cells to the otherwise lethal hypoxic microenvironment.

Although IGF2 was up-regulated by hypoxic stress and can promote angiogenesis through the IGF2 receptor in vascular endothelial cells, miR-483-5p, which is processed from IGF2 intron 2, was down-regulated. It acts as an endogenous angiogenesis inhibiting factor, at least in part through targeting serum response factor (SRF), which is required for angiogenesis [34]. Up-regulation of miR-483-5p correlates with the progression of human lung adenocarcinoma. MiR-483 is activated by the Wnt-β catenin signaling pathway and directly targets two putative metastatic suppressor proteins, Rho GDP dissociation inhibitor alpha (RhoGDI1) and activated leukocyte cell adhesion molecule (ALCAM) to promote epithelial to mesenchymal transition (EMT) [35].

An H19-related miRNA is miR-675, which is processed from H19 exon 1 [18]. Hypoxia up-regulated the expression level of miR-675 along with H19 RNA. We recently reported that under hypoxic stress miR-675 was elevated and so promoted the EMT process [36]. Furthermore, in hypoxic human articular chondrocytes, miR-675 is induced in a Sox-9 dependent manner and promotes the induction of Col2aI cartilage matrix gene [37].

Encoding miR-675 is one of the important roles of H19 RNA. MiR-675 is expressed exclusively in the placenta from the gestational time point at which placental growth normally ceases. The physiological role for miR-675 is to limit placental overgrowth, at least in part by targeting igf1r [38]. In cancer, a seemingly contradictory function is attributed to miR-675. In colorectal cancer cells for example, miR-675 targets retinoblastoma (RB) so boosting cellular proliferation [39]. In human gastric cancer miR-675 enhances cell proliferation by targeting the tumor suppressor RUNX1 [40]. It remains to be established how miR-675 target selection is accomplished in both the physiological and the pathological state.

We recently showed that miR-675 is important in the up-regulation of Slug expression through an unknown target. E-cadherin expression is abolished, resulting in the induction of EMT in many cancer types including lung cancer [36]. These findings are in agreement with the report that miR-675 promotes invasion in glioma cells by directly targeting CDH13 [41]. CDH13 is a tumor suppressor gene and its down-regulation is important step in the progression of lung cancer [42].

Recently, Wang *et al.* analyzed miRNA profiles in lung cancer patients. In this study they found that miR-675 was up-regulated significantly and has a major role in lung cancer through its regulation of its target genes, in particular RB1 [43].

Perhaps the best understood role in hypoxic stress is that of miR-210. It was shown that HIF-1 induction of miR-210 stabilizes HIF-1 through a positive regulatory loop. MiR-210 is up-regulated in lung cancer as well as in other types of cancers and over-expressed at late stages of NSCLC [44]. Moreover, miR-210 increases the resistance of hypoxic lung cancer cells to radiotherapy through HIF-1 and may be involved in promoting efficient genomic double strand breaks [45]. Validated targets of miR-210 include genes functioning in various aspects of tumorigenesis including cell cycle regulation and apoptosis, DNA damage repair, angiogenesis mitochondrial metabolism and antitumor immune response [46]. Its role in cancer is under debate as well as its prognostic impact [47,48].

### H19 lncRNA gene and human cancers: a focus on lung cancer

*H19* was the first lncRNA gene discovered [49] and is paternally imprinted. In humans, *H19* maps to chromosome 11p15.5, in close proximity to the reciprocally imprinted *IGF2* gene. It transcribes a 2.3 KB non-coding RNA transcript by RNA polymerase II and splices to five exons. Alternatively spliced variants have been also reported, however the significance of this alternative splicing is still unknown. One such variant was reported by our group to have restricted expression in embryonic tissues but not neoplastic ones [50]. H19 is highly expressed throughout development of the embryo and fetus, and also to the large extent in the placenta, but is shut down in most tissues shortly after birth.

In recent years it has become increasingly clear that the *H19* gene locus is far more complicated than initially thought. H19 opposite tumor suppressor (HOTS), a protein coded by the H19 antisense transcript and that has tumor suppressor activity has been described recently. Another relatively very long (~120 KB) and short lived transcript called 91H spans the *H19* gene locus and possesses tumor promoting activity, and is transcribed also in an antisense direction. Furthermore, the H19 transcript itself gives rise to the oncomir miR-675 which is processed from its first exon. The relations between these different products and how they are deregulated in the context of tumorigenesis remain to be elucidated (for review see [51]).

The principal characteristics of imprinted genes are their onco-fetal behavior pattern and their uniparental mono allelic expression [52]. In several human cancers, both pediatric and adult, release of imprinting underlies gene expression. This alteration was identified as a loss of imprinting (LOI). H19 lncRNA can be over-expressed via the LOI mechanism. This was found in a variety of malignancies, but not all. The significance of *H19* LOI in cancer is not well understood.

*H19* expression is induced by chemical carcinogens, retinoic acid, steroid and peptide hormones, growth factors such as hepatocyte growth factor (HGF), tissue repair factors, cytokines, and hypoxic stress [51].

Furthermore, we and others have identified various transcription factors such as E2F [53], HIF-1α [54], Slug [36], p53 [54,55] and c-myc [56] that can modulate H19 gene expression. Dysregulated activity of these transcription factors can affect many aspects of tumor biology including lung tumorigenesis.

**Figure 1:**
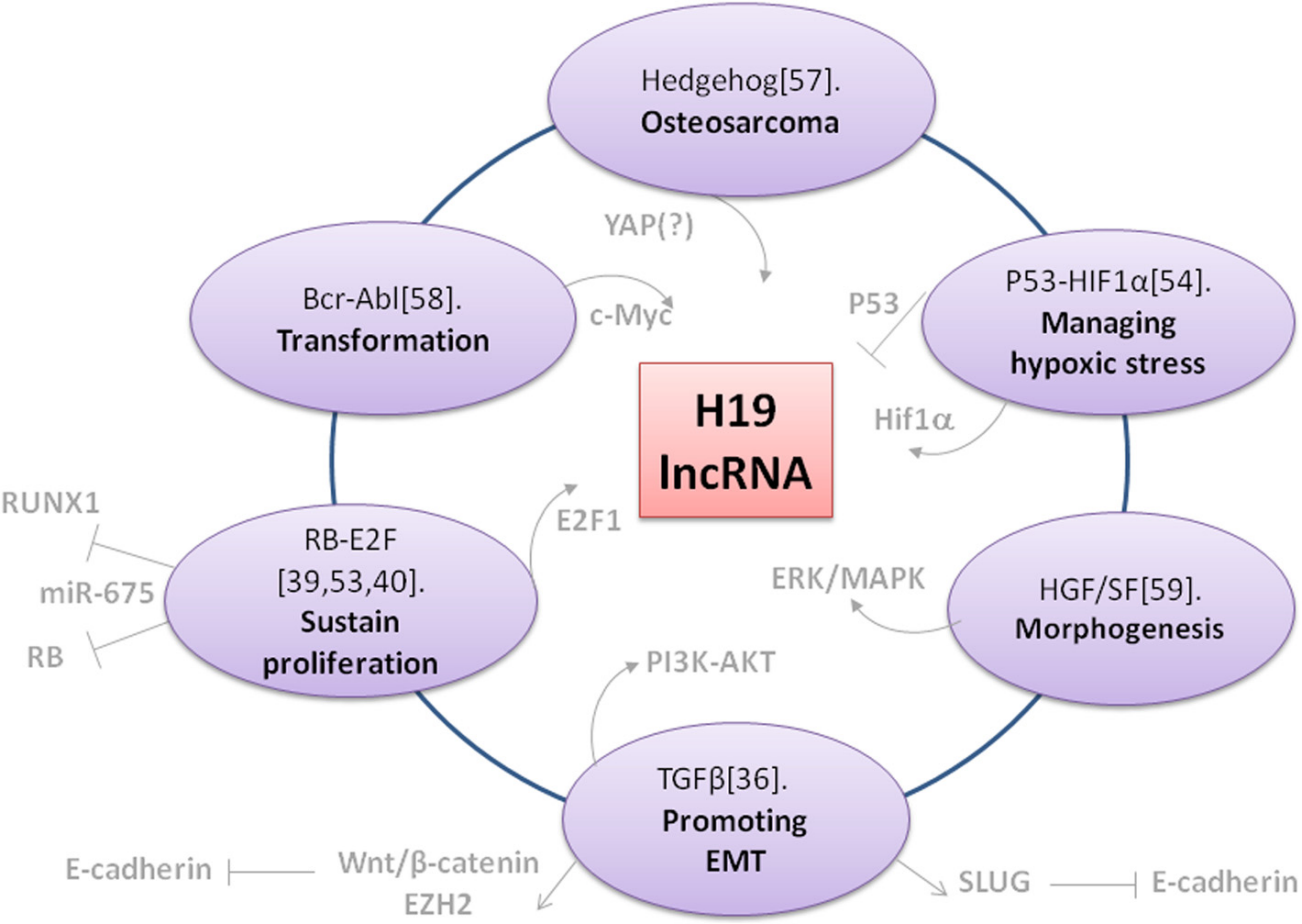
**Validated signaling pathways that modulate H19 lncRNA expression.** Shown are different signaling pathways reported recently that modulate the H19 lncRNA expression. Inside the circle are upstream transcription factors and signal transducers that modulate H19 lncRNA expression. Outside the circle are the downstream targets of either H19/miR-675. These signaling pathways play different fundamental roles in tumor biology including lung tumorigenesis.

Recent reports have linked H19 lncRNA to various fundamental pathways known to be involved in tumorigenesis, including the p53 and HIF-1α [54], hedgehog [57], TGF-β [36], Bcr-Abl [58], RB-E2F1 [39,40,53], HGF (MET) [59] and Wnt/β-catenin signaling pathways (Figure 1). The Wnt/β-catenin pathway is activated by H19 lncRNA when associated with enhancer of zeste homolog 2 (EZH2), and this resulted in down-regulation of E-cadherin [60].

**Figure 2:**
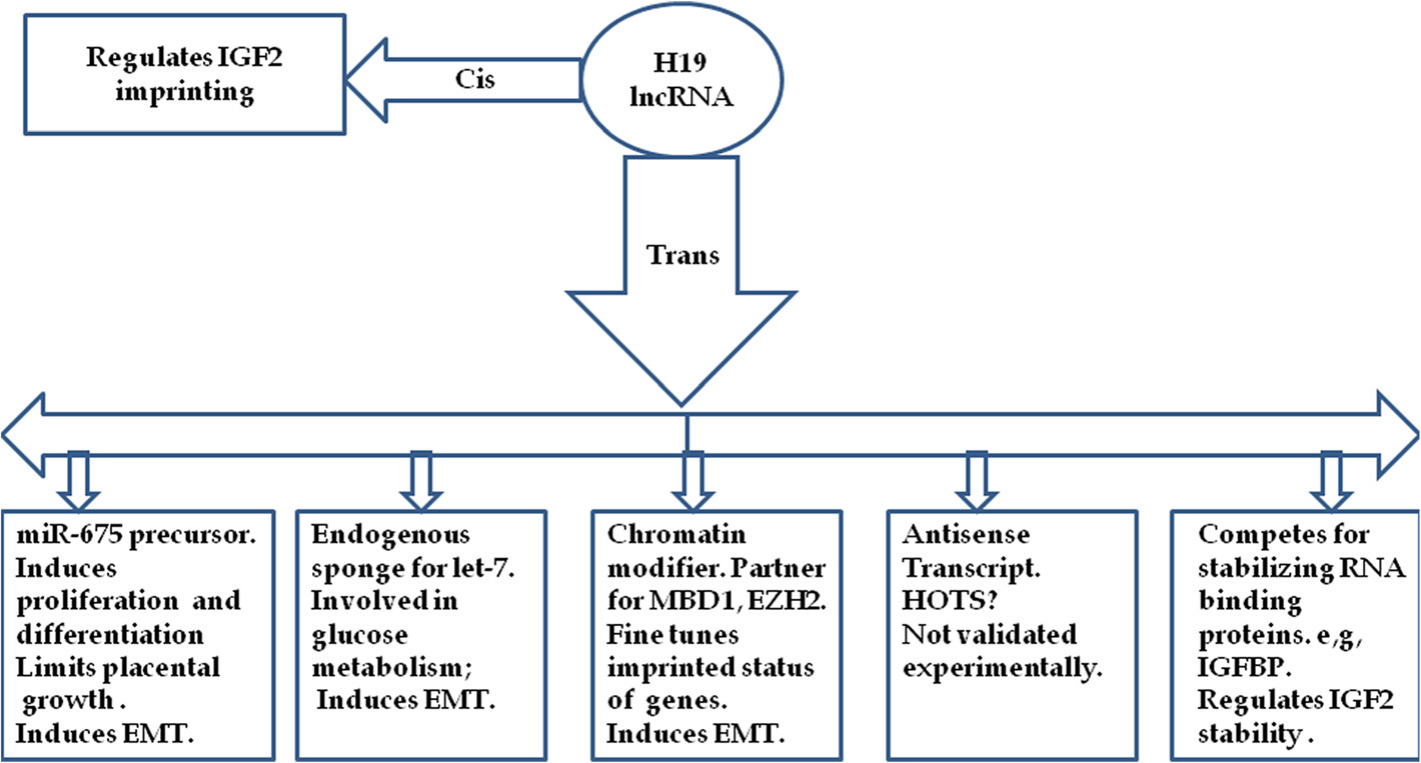
**Validated/possible patterns through which H19 lncRNA can perform its function.** The H19 gene can function in *cis* to control the imprinting of IGF2 by competing for common enhancers located downstream of H19 gene. In *trans* H19 lncRNA was reported to function through various patterns illustrated above. The possibility that H19 can function as an antisense transcript for HOTS is still not proven.

Furthermore it was shown that H19 lncRNA can modulate chromatin structure within the imprinted gene network through association with methyl-CpG-binding domain protein 1 (MBD1) [23]. Recently, H19 lncRNA was demonstrated to function as an endogenous sponge for miRNA [19]. It was further shown that H19 RNA can function as a molecular sponge for let-7 tumor suppressor miRNA, as it harbors both canonical and non-canonical binding sites for the let-7 family of miRNAs [61]. Consequently, H19 relieves let-7′s inhibitory effect on the oncogene Hmga2 [62]. H19 lncRNA is thus emerging as one of the key players in cancer biology. Figure 2 summarizes various validated and possible modes through which H19 lncRNA can function [18,23,36,39-41,60-66].

We have identified hypoxia as a principal inducer of H19 lncRNA [54,67]. H19 lncRNA functions, consequently, as a stress modulator and a survival factor and is involved in several fundamental processes triggered by hypoxia including EMT, metastasis and neo-angiogenesis [36,54,68]. EMT is an important process on the way to the malignant phenotype; notably H19 up-regulation occurs in the stroma as well as in the epithelium [69]. In the metastatic tumor stage, which bears a striking similarity to the embryonic stage, H19 involvement appears to be essential: adherent and cohesive cells lose their anchorage, migrate under stressful conditions to remote sites and replicate with neovascular support.

In lung cancer cells, H19 is induced upon hypoxic stress in a p53-dependent manner. Reconstitution of a wild type p53 in lung cancer cells possessing a null p53 mutation, abrogates H19 induction by hypoxia. The effect of p53 is nuclear but independent of its tetramerization, which is needed for its transcriptional activity. Knocking down H19 lncRNA in hypoxia inhibits lung cancer cell proliferation. For lung cancer cells possessing wild type p53, simultaneous inhibition of p53 and overexpression of HIF1-α were needed to upregulate H19 lncRNA significantly upon hypoxia [54].

In an attempt to understand the oncogenic role of H19 lncRNA in lung cancer, we utilized siRNA-mediated knockdown of H19. This approach resulted in very significant suppression of anchorage-independent growth, as assessed by colony formation in soft agar, implying that H19 lncRNA is functioning as an oncogene [36,67]. Most importantly, we provided strong evidence showing that the tumorigenic and scattering effect of HGF/SF on the A549 lung carcinoma cells can be attenuated by H19 knockdown [36]. Both HGF and its receptor, the MET tyrosine kinase, are overexpressed in lung cancer and as such are targets for therapeutic interventions. Several compounds against MET/HGF are in clinical trial and have been shown to be active against lung cancer (for review see [70]).

Furthermore, we recently provided evidence that H19 lncRNA significantly promotes the metastasis of lung cancer cells *in vivo*. This could be explained at least in part by the ability of H19 lncRNA to suppress the expression of E-cadherin and thus promote the EMT process by up-regulating Slug expression through a mechanism that involves miR-675 [36].

Further support for the involvement of H19 in lung cancer can be found in other reports. It was revealed that H19 lncRNA is over-expressed in lung cancer due to the loss of imprinting [71]. In addition, we reported that various cancers that metastasized to the lung show high expression levels of H19 lncRNA [36].

A correlation between the expression level of H19 and c-Myc has been found in a panel of 240 NSCLC tumors. c-Myc binds to evolutionarily conserved E-boxes near the imprinting control region to facilitate histone acetylation and transcriptional initiation of the *H19* promoter. It was deduced from this study that c-Myc induction of H19 plays an important role in transformation process [56].

Strong support for the linkage between lung cancer development and H19 lncRNA expression was provided by a study that revealed that smokers have dramatically elevated H19 lncRNA levels without alternation of the expression levels of other imprinted genes in the cluster. The up-regulation of H19 was monoallelic and it was suggested that overexpression and eventual LOI of *H19* may represent early markers in the progression of airway epithelium toward lung cancer [72].

Recently it was revealed that mineral dust-induced gene (mdig) enhances H19 expression by influencing the heterochromatin structure through reduction in the level of H3K9me3 in the promoter as well as ICR region of the *H19* gene in adenocarcinoma lung cancer cells [73]. Lung cancer patients with high expression levels of mdig and H19 manifest poorer survival. Initially mdig was identified in people exposed to mineral dust in the mining industry and have chronic lung disease.

### Other lncRNA of the H19-IGF2 axis

Two other lncRNA transcripts that are antisense to both *H19* and *IGF2* have been documented. 91H is a long and relatively unstable lncRNA which spans *H19* in antisense direction and is over-expressed in breast cancer [74]. It contributes to *IGF2* activation in *trans* and promotes tumor metastasis and predicts poor prognosis in colorectal cancer [75]. *IGF2-AS* is transcribed antisense to *IGF2*. This transcript is also maternally imprinted and overexpressed in Wilms’ tumor [76]. The roles of both transcripts in lung cancer have yet to be elucidated.

### H19 and mir-675 as tools for diagnosis and prognosis in lung cancer

To increase the survival of lung cancer patients we need to improve the sensitivity of the diagnostic methods; the earlier the diagnosis, the more effective the treatment. In general, the development of molecular biology methods has improved the early detection of malignancy. These methods also aid in providing a more accurate prognosis and in choosing the best treatment options. These advantages are also valid for lung malignancies.

The molecular characterization of lung tumors has increased the available options for noninvasive methods of diagnosis. Over the last decade the use of body fluids for lung tumor diagnosis has become common. This resulted in significant decrease of the usage of invasive methods.

Lung cancer is one of types of cancer in which many lncRNAs or miRNA molecules are found in blood (plasma/serum), free or inside exosomes [77-79]. In addition many studies have searched for a miRNA in sputum that is specific to lung cancer patients [80,81]. The high expression of H19 and miR-675 in lung tumors suggest that they could be used to detect lung cancer in fluid body like sputum (this has not yet been attempted). However, miR-675 has been recently shown to be secreted via the exosome [82]. The diagnostic and prognostic potential needs to be evaluated.

### Therapeutic implications

**Figure 3:**
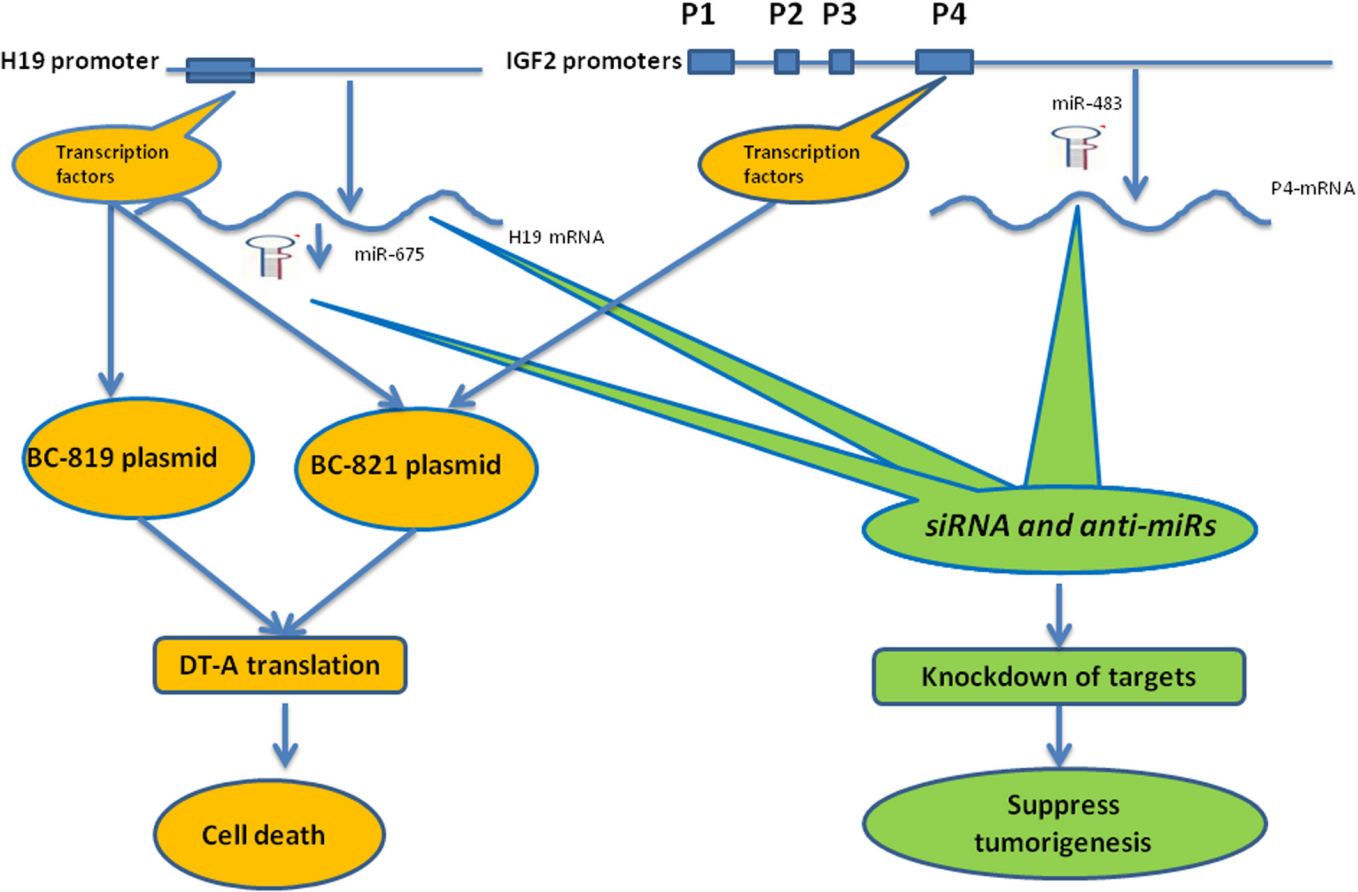
**Different therapeutic approaches based on H19-IGF2 gene loci for the treatment options of lung tumorigenesis.** Different therapeutic approaches can be harnessed to target specifically lung cancer cells with aberrant H19/miR-675 and IGF2/miR483 expressions. (Left): Transcriptional targeting approach utilizes transcription factors that activate the expression of the endogenous H19 lncRNA to drive the expression of a cytotoxic gene (DT-A) from the therapeutic plasmid vector (BC-819) in a manner specific for tumor expressing H19 gene products. BC-821 therapeutic vector in addition to H19-DT-A cassette contains another therapeutic cassette that drive the expression of DT-A under the transcriptional control of P4-IGf2 promoter. Thus BC-821 would increase both the cell killing capacity and the therapeutic index. (Right): Different cancer specific transcriptional products of the *H19-IGF2* loci ( e.g. H19, miR-675, P4-IGF2, miR-483), can be specifically knocked down alone or in combinations utilizing siRNA and antisense technologies. The phenotype predicted is retardation of lung cancer growth and progression depending on the targeted product.

A. Targeted personalized medicine
  H19 is commonly expressed in human lung cancer, but a given percentage of the patients are expected to be negative. The first stage should be the exclusion of H19 negative patients. We have gained relevant experience from the treatment of bladder transitional cell cancer (TCC) patients, where a phase I/IIB clinical trial has already been completed [83]. Patient selection is achieved using labeled H19 riboprobe in-situ hybridization of tumor samples. Expression intensity is quantified by a computerized histogram. Normal epithelia and the tumor carcinomatous as well as its stromal components can easily be distinguished. Exfoliated cells may also be informative using this technique; however, good-size tumor samples are usually available from TCC patients, which is not the case with bronchial biopsies. An alternative diagnostic tool, which can cope with tiny samples, is RT-PCR, which allows the accurate measurement of H19 transcript levels.
B. Therapeutic agents
  Therapies that target different cancer specific transcriptional products of the *H19-IGF2* loci or that exploit its transcriptional regulatory elements to drive the expression of a cytotoxic gene specifically to tumor cells would be critically advantageous over the current conventional therapeutic drugs, since they spare normal cells. Lack of tumor specificity is mostly responsible for the adverse events caused by the conventional drugs, which limits greatly the therapeutic doses. Expression of a DNA-based therapeutic toxin transcriptionally driven by *IGF2-P4* or *H19* promoters is predicted to occur in a manner specific for tumor expressing these gene products. However, the potential of this transcriptional targeting approach might be even greater if the cancer-specific promoters *H19, IGF2-P3, and IGF2- P4*, are utilized in the same therapeutic construct because many tumors that lack H19 gene expression have been found to express IGF2. The different therapeutic approaches discussed below is summarized in (Figure 3). Furthermore, the nature, advantage and disadvantage of these therapeutic approaches are summarized in Table 1.

**Table 1:** **Comparison between different possible therapeutic agents for lung cancer**

	**BC-819**	**BC-821**	siRNA*	Anti-miR*
**Nature**	Plasmid DNA.	Plasmid DNA.	Double stranded RNA.	Antisense RNA.
**Targeted therapy**	Yes.	Yes.	Yes.	Yes.
**Stability**	Relatively stable in serum [83-85].	Relatively stable in serum [87,89,90].	Rapidly degraded by serum nuclease.	Rapidly degraded by serum nuclease.
**Safety**	Mild safety problems [83-85].	Safe in animal model. In human subject ND.	ND	ND
**Homogeneity**	Very high	Very high	Depends on manufacturing process.	Depends on manufacturing process.
**Pharmacokinetics**	Well established [51,83].	Well established.	ND	ND
**Mode of action**	Suppresses translation initiation specifically in cancer cells leading to cell death.	Suppresses translation initiation specifically in cancer cells leading to cell death.	Knockdown target gene mRNA depends on intrinsic RNAI machinery.	Knockdown target microRNA level depends on complementary base pairing.
**Combinational therapy**	Can be given alone or in combination with conventional drugs.	Can be given alone or in combination with conventional drugs.	Can be given alone or in combination with conventional drugs.	Can be given alone or in combination with conventional drugs.
**Transfection efficiency**	Low.	Low. Bigger plasmid size expected to have lower transfection efficiency.	High.	ND.
**Therapeutic index**	High	Very high	High	ND.
**Costs**	Relatively low	Relatively low	Relatively high	Relatively high

ND: Not determined.

* Could be engineered through DNA plasmid vectors and thus have mostly the characteristics of plasmid vectors.

The table summarizes different aspects related to the therapeutic tools showing advantages and disadvantages of each tool where possible.

I. The BC-819 plasmid.
  BC-819 is a plasmid vector carrying the *H19* promoter, to drive the expression of the A chain of diphtheria toxin (*DT-A*), which inflicts damage to the cells expressing H19 lncRNA specifically by inhibition of protein synthesis. This engineered toxin is devoid of the *DT-B* fragment, thus cannot reenter neighboring cells. Following pre-clinical animal model testing in various cancer models (such as bladder, ovarian, and pancreatic colorectal hepatic metastasis), clinical trials at various phases of cancer progression have already been completed.
  The drug safety profile was excellent. No serious adverse events related to the study drug were observed. Toxicity was primarily local and resolved without sequelae. Subsequently, BC-819 has been given on compassionate basis to a limited number of patients afflicted with colorectal hepatic metastases, pancreatic cancer and relapsed ovarian ascitic carcinoma. All these cases had no untoward toxicity and were clinically beneficial (for reviews see [51,84,85]).
  The therapeutic potential of BC-819 for the treatment of lung cancer in mouse tumor models was investigated by our group. BC-819 was very efficient (>90%) in reducing lung cancer cell growth *in vitro. In vivo*, aerosolized PEI/BC-819 is capable of reducing growth only in tumors arising from the luminal part of the airways that are therefore directly accessible for inhaled BC-819 [86].
II. The BC-821 plasmid.
  BC-821 is a double promoter (*H19* and *IGF2-P4*) DT-A-expressing therapeutic vector. Here the approach is to create a new family of plasmids regulated by two regulatory sequences, which in their natural genome position are both proximately located and are reciprocally imprinted [87]. The combined expression levels of H19 lncRNA and IGF2 driven by P4 promoter in lung cancer patients is higher than each one alone, so the use of this vector should substantially increase the therapeutic index.
  *In vitro* as well as *in vivo* studies demonstrated that the double promoter vectors exhibited superior activity compared to the single promoter vectors in many cancer models [88-90] including that of the lung (unpublished data).
III. siRNA and anti-miRs.
  This is a relatively new entry into the antineoplastic arsenal. Double-stranded, small interfering RNA (siRNA) molecules can specifically ablate the expression of genes that share their sequence. Libraries containing siRNA molecules that target all genes in the human genome are now available. Pre-clinically, siRNA molecules have been tested in a NSCLC cell line, to suppress genes responsible for drug resistance [91]. Furthermore combination of EGFR inhibitory agents with EGFR-specific siRNA increases the therapeutic efficacy in NSCLC [92]. In this context, H19-targeted siRNA abrogated H19-induced multi-drug resistance, via its involvment in the regulation of MDR1 promoter methylation, whereas H19 knockdown suppressed MDR1/P-glycoprotein expression [93]. Furthermore we recently showed that EMT induced multi-drug resistance is accompanied by an increase in H19 expression [36]. In our laboratory, siRNA-H19 suppressed anchorage independent growth of lung cancer cells and also suppressed xenograft bladder cancer (UMUC3) as well as hepatoma (Hep3B) growth [36,67]. A potential alternative target could be IGF2 which may be present in H19-negative cases, for whom siRNA-IGF2 could be used. It is also conceivable that dual targeting of both the H19 and IGF2 genes could be employed.
  The lung is an attractive target organ for siRNA-based therapy. While delivery approaches could be accomplished via systemic (e.g. intravenous) or pulmonary routes (intranasal, intratracheal or inhalation), the latter is preferable as it allows higher retention of siRNA in the lung tissues and also reduces the risks of systemic toxicity. Large efforts have been devoted to establish effective siRNA nanocarriers for direct pulmonary delivery [94-96]. The oncogenic activities of both miR-675 and miR-483 in lung cancer represent further therapeutic opportunities whereby antisense oligonucleotides that can bind directly to these miRNAs can block their activity. This anti-miR approach was found to be effective in pre-clinical studies for lung cancer [97-99].

### Summary and future perspectives

It is becoming increasingly clear that H19 and miR-675 are key players in many human cancer types including those of the lung. H19/miR-675 function can be attributed to various fundamental aspects of tumorigenesis,ctoincluding hypoxic stress response, angiogenesis, EMT, metastasis and multi-drug resistance. The past five years have witnessed an unprecedented increase in scientific papers linking H19/miR675 to fundamental signaling pathways known to have a pivotal impact on tumorigenesis, including the hedgehog, p53, HIF-1α, TGF-β, bcR-abl, and Wnt/β-catenin signaling pathways. Especially notable is that over the last two years more than ten reports have been published that linked H19/miR-675 to EMT and the metastasis process.

Despite this progress, still many questions are left to be answered. The *H19* gene locus is far more complex than initially thought. Although it is clear that some of *H19* gene functions are performed through miR-675, we still need to explore what functions can be performed by H19 that do not involve its miRNA. miR-675 is processed from H19 lncRNA, but both are up-regulated in many types of cancers and both can be up-regulated by common triggers. It remains unclear how this is accomplished, taking into consideration that miR-675 is processed at the expense of H19 lncRNA. One option is that *miR-675* could have its own regulatory sequence, beside being processed from H19. This has not been investigated yet. Furthermore the interplay between the various products processed from the H19 gene locus remains largely unknown.

Whether H19 and miR-675 are secreted in the exosomes/body fluids and can be used as diagnostic as well as prognostic tools in lung cancer patients needs to be elucidated.

The development of DNA-based therapy involving H19/miR-675 is underway in our lab, and show promising results in preclinical models of lung cancer. This will pave the way for phase I clinical trials in lung cancer hopefully in the near future.
